# Abstract of Proceedings of Chicago Medical Society

**Published:** 1860-02

**Authors:** 


### ABSTRACT OF PROCEEDINGS OF THE CHICAGO MEDICAL SOCIETY.

The society met at the residence of Dr. W. W. Allport, Tuesday Eve., Dec. 29, Dr. Waite in the chair.

Members present: Drs. Allport, Byford, Wickersham, Heydoch, Graham, Fisher, Powel, Faircloth, Isham, Andrews, Deville, Holmes, Davis and Steele. Visitors: Prof. Allen, Rea and Dr. Mott.

The Sanitary Committee on request, begged leave on the part of Dr. S. Wickersham, of the South Division, to report the following:

"The diseases with which we have met, have been those of the corresponding months of other years. In our July report, we stated that about the usual number of children are suffering with the disease peculiar to the season. Many of these have gone to their final home, others are stout and healthy, and a small percentage are still the victims of chronic Diarrhœa, or secondary disturbances which prevent a return of the healthy aspect, and joyful laugh. Another seasons experience has but added to our conviction, that in these cases we should beware of medication and trust more confidently to hygienic measures, give increased attention to dress, aliment, air and bathing. By a judicious administration of medicines, much may be accomplished, but let them be dealt out with caution, and ever remembering that attention to the above is a *sine qua non* to a successful treatment. Without pausing to detail treatment, I would simply state that with those cases of chronic diarrhœa and emaciation with which we so often meet at this season of the year, there is no simple remedy upon which I place more reliance, than the tinctura cinchonæ comp. This remedy in most cases given in doses of from 20 gtts to a drachm will be found very beneficial.

Comparatively but few cases of dysentery have occurred during the season. I believe it was the most prevalent about the middle of September. It will be remembered that about the 10th, we had copious showers, which was the termination of the dry weather. Immediately succeeding the rain we saw a large additional number of cases of dysentery and remittent fever, and these of a more obstinate type than those which we had previously seen. Until this period I had seen no case of dysentery that gave me any uneasiness, but in the interval between the 10th and 25th, I met with several cases that gave me some anxiety, and required additional care in the treatment. I lost but one adult, I had his disease controlled at two separate periods, but from violation of my instruction, in eating fruit etc., he passed into his second relapse which proved fatal. Remittent fever was not prevalent until after the September rain, when in two days immediately succeeding the rain, I took thirteen cases under treatment, showing that nothing was wanting for the production of the disease but moisture. These in most instances were easily managed. We saw no case if not already arrested, but what was converted in a very few days into an intermittent. Our plan of treatment is to relieve the bowels, to obtain by diaphoretics, a distinct remission, and administer an anti-periodic.

Intermittent fever in other years that we have practiced in Chicago, has prevailed to a considerable extent along the southern boundary of the city, more particularly to the part adjacent to the river. Not so this. There have been isolated cases throughout the south division, but the majority have been connected outside of the city. We have never seen but one case and that a recent one, that was not easily arrested by the quinine sulphas, after properly preparing the system for its reception. It was the case of a girl about fourteen years of age, who I had under treatment in September. It was at first a remittent. After three days the intermission was marked. The sulphate of quinine was given in the intermission, at first in moderate doses, failing to arrest it, it was increased so as to produce its characteristic effect upon the brain, and was sustained for several days, during which it was arrested for one day only. The fever came on every evening about 8 o'clock. Prof. Wood says that he has never seen but some three cases, that the quinine sulphas did not arrest, and those when a young practitioner, and believes that he did not give enough of the remedy, but had he seen this case, I think he would have been satisfied that he had seen a case in his older days that the remedy would not effect. I then administered the liquor potassae arsenitis in eight drop doses three times each day, this was given about a week, when odema about the lower eyelids was perceptible. The system being now sufficiently under its influence, and it being necessary to suspend the remedy, and as no impression apparently had been made upon the disease, I was at a loss to know what to select amongst the many reported articles.

At this juncture, meeting with my friend, Prof. N. S. Davis, I detailed the case to him. He told me he thought I had better administer the cornus florida, and I understood him to say that he was accustomed to give it when the sulphate of quinine failed. I should have followed his counsel, but when I returned the next day I learned that her attack escaped the previous night. No further medicine apart from some ferruginous preparation was given, and she has had no symptoms of a return. In this case the arsenic proved eminently superior to the quinine.

During the latter part of September, and early in October, cynanche parotidea or mumps, prevailed extensively in some isolated sections of the South division. In the district bounded by Twelfth on the North, and State on the West, these were of the most violent form that I have ever witnessed. I will not weary you by a detail of the symptoms and treatment, but will give you an idea of the violence of them, by saying that in three children the parotid suppurated, and in one of these the gland on both sides; and another succumbed while the gland was undergoing the suppurative process. I would say here that I was not mistaken in my diagnosis. This was not accompanied by any form of angina or diptheria. But it was pure and uncomplicated mumps. The throat was scarcely altered from health, and deglutition was attended with but slight pain. It is very rare for the gland to suppurate while affected with mumps. Prof. Wood says in his work on practice, that he does not remember to have witnessed a case where this has occurred. These were the first that I had seen. I lanced the glands as soon as the presence of pus was undoubted, and the quantity that was discharged for some days is scarcely credible. Active stimulation was required to support the strength while the gland continued to discharge, and it left the children very much debilitated. It is proper to say that I did not see these cases in time to adopt any effective means to prevent the suppuration.

From such knowledge as I have obtained from my medical friends, and my own personal knowledge, I am happy to be able to inform you that there have been but few cases of enteric fever, and these generally of a mild type, and terminating favorably a few days sooner than usual. The cases that we have seen have needed but little medicine. We view the disease as self limited, and think the physician has accomplished all that he can when he places and preserves the system in the most favorable condition for the elimination of the morbific matter upon which the fever depends.

We believe that a large number of our cases of typhoid fever of this region are not of that pure character that we meet with in the East. A very large proportion of the cases that I see are a mixture (especially in the early stages) of remittent and typhoid, hence we have our advocates of quinine, and much good will be accomplished by its administration in such cases, until that part of the affection which is not typhoid is overcome; your patient you deem convalescent, and perhaps leaves his bed for a period, but he is certain to return, and destined to pass through with an attack of typhoid fever, which, so far as the eradication of the disease is concerned, quinine is of no utility. We have recently seen the case of a lady where this malarious influence became manifest about every seventh day, and this throughout a six weeks attack of typhoid fever. The sulphate of quinia, as often as it recurred, was given with the most marked effect, indeed would completely arrest the intermittent fever coming on every other day. But we failed to observe the least amelioration of the typhoid symptoms during the administration of the antiperiodic. Hence we use it only as a stimulant in enteric fever.

Perhaps I should not close this report without a word in reference to diptheria. We believe that our people are unnecessarily frightened. We have seen but one case that deserved the appellation. We have seen several cases during the autumn of pseudo-membranous croup and kindred diseases, but the tendency among the "similia" school is to pronounce all these diptheria, and also in many instances picture this as a new and very dangerous affliction. To all of which we very respectfully beg to take issue, and express as our decided conviction that the cases of pure diptheria have been very few."

Also, Dr. Holmes, of the North Side, reported as follows: "The facts which I have been able to collect from my own observation and inquiries, regarding the sanitary condition of the North division, can scarcely add anything of interest to the statements in my last report. The people have still been blessed with almost unparalleled exemption from sickness. Although the last three autumns have been to a great extent free from severe epidemics and fatal disease, the present season, I am sure, has even been more favorable to health. Whatever may be said regarding this general good health of our city, and the comparatively small amount of business done by physicians and apothecaries during some of the months, it is true, I suppose, that the bills of mortality for corresponding months of the two or three years past do not differ to a very great extent; it is said the number of deaths continues nearly the same. How this is to be accounted for, whether by an increase of population or otherwise, I am unable to say.

In the neighborhood of the sands there has been considerable intermittent fever, principally however among those who have had the disease before. In other portions of the North division, also, I have seen a few cases. In the same locality I have met with typhoid fever. The type of disease in my practice, however, has not been in general typhoidal.

Sore throats, with swelling of uvula and tonsils, have been quite frequent; but I have not met with any case of diptheria, nor of scarlet fever, or measles.

One of my cases of typhoid fever I consider somewhat singular, and therefore will give a brief report. An Irish woman, aged 38, was taken sick immediately after the recovery of her husband, whom I had treated for intermittent fever. She had been complaining of weariness and fatigue for several days before I left her husband. She grew worse, and was obliged to keep her bed nearly all the time for a week before sending for me, although she did not feel very badly, unless she sat up. At this time I found her with all the symptoms of mild enteric fever. She did not complain of much uneasiness; rested quietly, and thought she would be about in a few days. The pulse was 96 and small. Tongue was not much coated, but red at the tip and along the edges. There had been considerable diarrhœa, with slight tenderness in right iliac region, but scarcely any tympanitis, perhaps no more than is often found in health. The general appearance and spirits of the patient were so good, that I only prescribed QUININE, with opium and potass. chlo. in the intervals. I allowed a little weak beef tea and gruel. The case continued for a week in a most favorable condition. All the symptoms had improved; the patient had gained so much strength that she wished to sit up. I discouraged anything of this kind. On the eighth day, at my usual visit, I found her in a state of complete collapse; extremities cold, lips livid, wrists almost pulseless, countenance anxious and pinched. The abdomen was not in the least tumid or painful on pressure. Patient still retained her consciousness to the fullest degree, and seemed to know the danger of her condition. In spite of all treatment and stimulants she continued to sink for thirty-six hours, at the end of which time she died. This case, I imagine, was an example of that flattering and yet dangerous form of typhoid fever, in which ulceration of the bowels perforates the tissues, with scarcely an appearance of danger till the state of collapse, suddenly, and when least expected, supervenes.

I recollect to have seen a similar case, under the care of a distinguished physician, in hospital practice; the patient was so far convalescent that she was able to walk about the ward; she had been able to sit up, during the whole of her sickness, a short time each day. She suddenly became worse, with symptoms like those above described, without pain or swelling of abdomen, and died in a few hours. A small perforation of the bowel was found at the autopsy.

I would here state that during the past six months, ending Nov. 1st, 112 patients have been under the care of the surgeons of the Chicago Charitable Eye and Ear Infirmary, making an aggregate of 227 patients who have been under treatment since its organization.

Diseases of nearly every description and stage of progress among the poorest classes which our city can produce have been treated."

An interesting discussion of the views contained in the sanitary reports was participated in by the members of the Society generally.

The question, "What were the characteristics of the fevers prevalent in this city during the past autumn?" being next in order, Dr. Davis remarked in substance as follows:

The question for discussion had reference to the "characteristic features" of such cases of fever as had been noticed in this city during the past summer and autumn.

From the latter part of July to the middle of October, cases of periodical fever, both of the intermittent and remittent forms, were of frequent occurrence, though at no time unusually prevalent. In those cases he had observed nothing peculiar, either in the symptoms or the treatment required. One case only had presented a pernicious or malignant aspect. This occurred in a native of Ireland, living on Mather St., near the south branch of the river. When visited, the extremities were cold: the lips livid; the eyes sunken; the breathing hurried and irregular; the pulse small, feeble and frequent; pain in the bowels, with frequent serous discharges tinged with blood; and extreme restlessness. All these symptoms had supervened suddenly, immediately preceded by chilliness and pains in the back and limbs, such as usually attend the commencement of a paroxysm of intermittent fever. From these symptoms he inferred that the patient was in the midst of a paroxysm of pernicious fever, with irritation of the mucous membranes; and prescribed large sinapisms of mustard over the epigastrium and spine; an enema of tinct. of opium ʒj, in half a tea cup full of cold water, to be used immediately after each evacuation from the bowels; and a solution of sulph. morph., 2 grs., bi carb. soda ʒj, in water ℥jv, of which a tea spoonful was to be given immediately after each effort at vomiting. He also directed the following powders, viz:

| | | |
|---|---|---|
| ℞ | Sulph. Quin. | 20 grs. |
| | Pulv. Opii, | 10 grs. |
| | Proto-Chloride Hydarg. | 10 grs. |

Mix, and divide into six powders, the first to be taken in one hour after the visit, and the remaining ones at intervals of two hours until all had been taken. The next day the patient was found free from fever and other severe symptoms. By keeping up a moderate antiperiodic influence of quinine, and due attention to the bowels for two or three days, the patient rapidly recovered.

In regard to continued fevers, he stated that cases had occurred, as usual, throughout the season; but that they became more frequent during the month of September.

The only peculiarities observed, worthy of mention, were that a larger proportion of the cases than usual presented the phenomena of typhus, as distinguished from typhoid or enteric fever; and that many of them were ushered in with a distinct chill, followed by daily exacerbations for three or four days. The last named peculiarity was so prominent in several cases as to cause attending physicians to mistake them for true malarious remittents, and to treat them with quinine in antiperiodic doses. He said such cases led to two inquiries of much practical importance. 1st, Are they really cases in which the two types of fever, periodical and continued, are blended together, or are they simple cases of typhus in which the febrile symptoms undergo greater diurnal changes than usual?

2d. If they are cases of simple or unmixed typhus, what are the symptoms by which we can distinguish them from cases of the true remittent type?

The question whether the different types of fever are capable of being blended together in the same individual has been ably discussed in two reports to the American Medical Association, the first by Dr. S. H. Dickson, of Charleston, S. C., one of the most eminent teachers of practical medicine in the profession; and the other by Dr. Pease, of Janesville, Wis.

Without attempting te review the facts and arguments relating to the question, he simply expressed his own opinion that the elementary morbid conditions were, in some respects, so different in the two forms of fever, as to preclude the idea that we could have the *active* symptoms of both mingled together in the same patient. Hence he regarded the cases of continued fever that had presented distinct exacerbations and remissions during the first three or four days of their progress, as only deviations from the ordinary course of symptoms, and not dependent on the admixture of any true malarious influence. If this is true, it is of great practical importance to make the proper diagnosis between such cases and true remittent fever. To do this, he said, it was evident that we must rely on other symptoms besides the exacerbations and apparent remissions; for these had been so strongly marked in the beginning of many of the cases, occurring since the middle of August, that they had been actually mistaken for genuine remittent cases by some of our best practitioners, and treated with full doses of quinine, until the subsequent progress of the cases compelled a correction of the error. He believed, however, that a careful attention to the expression of countenance, the color of the lips; the state of the tongue; the feelings in the head; and the mental condition of the patient, would enable the practitioner to avoid the mistake alluded to. He stated that in all the cases of continued fever which had come under his observation, whether characterized by daily exacerbations during the first few days or not, there was a dulness and heaviness in the expression of countenance; red and dry appearance of the prolabia: a feeling of giddiness or swimming in the head on attempting to assume the erect position after lying down; and an apparent reluctance on the part of the patient to converse cheerfully, or even to admit that he was much sick, which he had never seen in the early stages of periodical fever. Some have claimed to be aided in making the diagnosis by the action of remedies. Thus, if the case presented the semblance of periodicity in the febrile movements, quinine in anti-periodic doses was promptly administered. If it fully arrested the progress of the fever, the case was regarded as a true malarious fever. If, however, the anti-periodic, was followed by simple diminution of the exacerbations without interrupting the fever, it was then regarded as typhoid or typhus, and treated accordingly. This practice is founded on the assumption that the quinine is harmless in the early stage of continued fever, even when it exerts no apparent influence over the continuance of the disease. But he doubted the truthfulness of this assumption. Several years since his attention was arrested by the apparently injurious effect of five grain doses of quinine in the early stage of a case of continued fever; its administration having been followed by a stupor from which the patient never recovered. And the only fatal cases that had come under his observation, in private practice, during the past season, were in patients to whom from three to five grain doses of quinine had been administered during the first two days after the treatment was commenced, under the impression that they were cases of "bilious remittent fever." These cases, three in number, were so well calculated to illustrate the peculiarities of fevers of the past three months, that they were related in detail. We have space only for their more prominent features. The first was a man aged about 30 years, who was attacked with all the ordinary symptoms of an idiopathic fever, ushered in by chilliness, and continued with distinct exacerbations and remissions. A respectable physician was called in, who prescribed alteratives and anti-periodic doses of quinine, which seemed to arrest the exacerbations, and for two days the patient thought he was rapidly recovering. At the end of that time, however, he found himself unable to keep up, on account of a peculiar degree of swimming in the head, a constant fever, a dirty white coat upon the tongue inclining to be dry in the middle; a dull expression of countenance, and slight wandering of mind during the night. The latter symptom soon became more marked, so that the low muttering delirium was constant until the fatal result, which was in ten days after he was seen by the speaker. The symptoms during that time were those of a well marked typhus; there being neither diarrhœa nor tympanitic abdomen, and yet the case terminated suddenly from copious intestinal hemorrhage. The other two cases were so similar in all their features, except the mode of death, and the apparent effects of the quinine so nearly the same, that it is unnecessary to repeat the description. In each the immediate effect was to arrest the exacerbations to such a degree, that the attending physicians entertained no doubt of the propriety of its use. Yet in each, the mind first became very dull and taciturn, then entirely wandering, with well marked symptoms of a low grade of typhus Such results had led him to doubt, not only the beneficial effects of quinine in the early stage of continued fevers, but its harmlessness also.

On motion, the Society adjourned.

